# Relationship between sella turcica morphology and dentofacial anomaly – a three-dimensional analysis

**DOI:** 10.1186/s13005-025-00544-3

**Published:** 2025-11-03

**Authors:** Bernhard Wiechens, Annika N. Füllgrabe, Christian Dullin, Jonas Q. Schmid, Phillipp Brockmeyer, Philipp Meyer-Marcotty, Anja Quast

**Affiliations:** 1 Department of Orthodontics, University Medical Center Göttingen, Robert-Koch-Str. 40, 37075 Göttingen, Germany; 2 Institute for Clinical and Interventional Radiology, University Medical Center Göttingen, Robert-Koch-Str. 40, 37075 Göttingen, Germany; 3 Department of Orthodontics, University of Münster, Albert-Schweitzer-Campus 1, 48149 Münster, Germany; 4 Department of Oral and Maxillofacial Surgery, University Medical Center Göttingen, Robert-Koch-Str. 40, 37075 Göttingen, Germany

**Keywords:** Sella turcica volume, Jaw malformation, CBCT, CT, Micro-CT

## Abstract

**Background:**

This cohort study aimed to evaluate the relationship between sella turcica (ST) morphology and dentofacial anomalies three-dimensionally (3D).

**Methods:**

Cone beam computed tomograms (CBCTs) and computed tomography scans (CTs) of 90 adults (46 women; age 28.1 ± 11.3 years) were analyzed. Dentofacial anomalies were evaluated (1) sagittally regarding skeletal classes (Wits appraisal), (2) vertically regarding the inclination of the jaws (maxillomandibular plane angle), and (3) transversally regarding skeletal symmetry (menton deviation from midsagittal plane). Sella morphology was assessed (a) linearly by ST-length, -width, -height, -diameter, and position to nasofrontal suture (CB); and (b) volumetrically by total, anterior, and posterior volume (Vol_T, Vol-A, Vol_P). Five porcine skulls were scanned by CBCT, CT, and micro-CT for method validation.

**Results:**

CBCT and CT both underestimated the ST volume compared to the volume measured with micro-CT. ST morphology differed significantly between the skeletal classes. Individuals with skeletal class I showed higher ST length than participants with skeletal class II (*p* = .024). Vol_T and Vol_A were higher in class II than in class III (*p* = .047; *p* = .019). Vol_A correlated with the Wits appraisal (*r* = .337, *p* < .001). Vertical and transversal jaw relation showed no correlation with ST variables. ST-width and CB were significantly higher in men than women (*p* = .004; *p* < .001).

**Conclusion:**

3D-diagnostics of ST morphology enabled conclusions about underlying dentofacial anomalies. Vol_A seemed to be a relevant marker based on its correlation and class-specific volumes. Future research could help to identify this as prognostic factor of developing anomalies.

## Introduction

The sella turcica (ST) plays a pivotal role in cephalometric analysis due to its radiographic stability and anatomical consistency [1–3]. As the maxilla and mandible show high transformation and dimensional changes during growth and development, a fixed cranial landmark is essential to reliably evaluate their relation [1, 3, 4]. Therefore, the ST holds a special position as reference and construction point [5]. Furthermore, it encloses the pituitary gland, which affects both the size and morphology of the ST as well as overall somatic growth. Conversely, morphological changes can indicate pituitary diseases such as tumors and hypoplasia showing its high systemic relevance [6–9].

In the systemically healthy population, possible factors that may affect the structural characteristics of the ST include genetic, developmental, and hormonal components. For example, ethnicity, gender and age are discussed to correlate with variations in ST dimension and shape [10–13]. Beyond these individual factors, increasing attention has also been directed toward the relationship between ST morphology and overall craniofacial anomalies, including dentofacial anomalies and impacted teeth. For instance, ST bridging occurs more frequently in subjects with palatal canine impaction than in controls [14] and patients with skeletal class III show significant higher rates of ST bridging than class I patient [15]. These associations suggest that morphological deviations of the ST may serve as indirect indicators of disturbed craniofacial development and contribute to early identification of underlying skeletal or dental irregularities.

However, the findings regarding the relationship between ST characteristics and craniofacial morphology remain controversial. While some authors describe significant differences in length and anteroposterior diameter of ST between the skeletal classes [16], others denied differences in ST diameter [17].

These inconsistent findings may be caused by different imaging techniques. The gold standard for cephalometric analysis remains the two-dimensional (2D) lateral cephalogram. Consequently, most available data on ST morphology relies on 2D imaging. One reason for this is the justifying indication for imaging, as well as the lack of cephalometric standards for three-dimensional (3D) assessment using cone beam computed tomography (CBCT) or computed tomography (CT). However, a 2D radiograph provides a flattened image of a 3D structure, leading to distortions and overlaps that can obscure anatomical details. In contrast, 3D imaging allows for precise visualization of depth, width and volume [18]. This level of detail is particularly important given the wide range of factors influencing the morphology of ST. Nevertheless, it has to be taken into account that morphometric data derived from CTs or CBCTs are subject to limitations in spatial resolution, contrast, and segmentation accuracy. Compared to micro-computed tomography (micro-CT), which is considered the gold standard for ex vivo imaging, clinical CT/CBCT do not necessarily reflect true anatomical dimensions but must be regarded as estimations. Larger voxel sizes, partial volume effects, and imaging artifacts can lead to deviations between the measured and actual morphology [19].

Therefore, the aim of this study was (1) to assess the extent to which CT/ CBCT imaging can reliably represent ST morphology, and (2) to investigate possible correlations between ST characteristics and the craniofacial skeletal configuration in adult patients with pronounced dentofacial anomalies in all three dimensions.

## Methods

This cohort study was conducted in accordance with the principles of the Declaration of Helsinki and was approved by the ethics committees of the University Medical Center Goettingen (No. 7/1/16). It complies with the STROBE-statement (Strengthening the reporting of observational studies in epidemiology) [20]. All participants provided written informed consent to participate in this study.

Residual biological material from the dissection was disposed of in accordance with the Animal Welfare Act via the University Medical Center Goettingen. The animals investigated were not euthanized for this study. Two skulls originated from meat production and were defined as slaughter material. Three skulls were provided to this study after euthanasia of test animals, which was required following an ethically correct series of experiments conducted by the Department for Oral and Maxillofacial Surgery at the University Medical Center Goettingen.

### Patients

90 participants (46 women; mean age: 28.1 ± 11.3 years) who received CT or CBCT imaging at the Department of Oral and Maxillofacial Surgery or the Department of Orthodontics at the University Medical Center Goettingen were included in this cohort study. All data were collected from February 2021 to July 2023 and were analysed twice by two independent investigators. Inclusion criteria were the availability of CT or CBCT imaging with a metric reference scale, full visualization of the viscerocranium and the cranial base in closed-mouth position, adequate depiction of the ST and bilateral occlusal support in the molar region. Exclusion criteria were fractures of the viscerocranium except the midface, head and neck oncological diseases, craniofacial malformations such as cleft lip and palate, congenital jaw anomalies or previous orthognathic surgery.

Patients were categorized in the sagittal dimension according to the Wits appraisal (skeletal class I: Wits − 2 to + 2 mm, skeletal class II: Wits > 2 mm, skeletal class III: Wits < -2 mm) [21]. The vertical classification was based on the maxillomandibular plane angle (neutral: ML-NL = 20.5–26.5°, hypodivergent: ML-NL < 20.5°, hyperdivergent: ML NL > 26.5° [22]). Transverse asymmetries were determined by menton deviation to the mid-sagittal plane (symmetric: Me-MSP ≤ 2 mm; asymmetric: Me-MSP > 2 mm) [23]. The demographic and clinical characteristics of the participants are presented in Table 1.

**Table 1:** Demographic and clinical characteristics of participants

Participants	*N* = 90
men	*n* = 44
women	*n* = 46
participants’ age	*M* = 28.1 years; *SD* = 11.3 years
participants by skeletal class	
skeletal Class I (Wits = [-2; 2 mm])	*n* = 30 (*M* = 0.5 mm; *SD* = 1.3 mm)
skeletal Class II (Wits > 2 mm)	*n* = 30 (*M* = 5.4 mm; *SD* = 3.0 mm)
skeletal Class III (Wits < -2 mm)	*n =* 30 (*M* = -8.9 mm; *SD* = 4.4 mm)
participants by vertical relation	
neutral (ML-NL = [20.5; 26.5°])	*n* = 28 (*M* = 23.4°; *SD* = 1.6°)
hypodivergent (ML-NL < 20.5°)	*n* = 30 (*M* = 16.6°; *SD* = 2.3°)
hyperdivergent (ML-NL > 26.5°)	*n* = 32 (*M* = 32.8°; *SD* = 5.1°)
participants by asymmetry	
symmetry (Me-MSP ≤ 2 mm)	*n* = 57 (*M* = 1.0 mm; *SD* = 0.6 mm)
moderate asymmetry (Me-MSP > 2 mm)	*n* = 33 (*M* = 4.2 mm; *SD* = 2.0 mm)

Presentation in means (M) and standard deviations (SD). Exclusion criteria: Participants with no skeletal malocclusion, previous orthognathic surgery, congenital craniofacial anomalies, cleft lip and palate, or syndromic conditions were excluded from the study

### Radiography

Twenty-eight CTs and 62 CBCTs were included in the present study. All data sets of CT imaging were acquired using the same device (Somatom Definition AS, Siemens Heathcare GmbH, Germany). Imaging was performed considering individual settings using 128-line multidetector acquisition, a slice thickness of 0.6 mm at 120 kV tube voltage, 650 mA tube current, an exposure time of 500 ms and a voxel size of 0.47 mm. All CBCTs were acquired using the same device (PaX Zenith 3D, Orange Dental, Germany). Imaging was performed in a field of view of 240 × 190 mm, a slice thickness of 0.3 mm at 120 kV tube voltage, 6 mA tube current, an exposure time of 2400 ms and a voxel size of 0.3 mm.

### 3D analysis

The linear dimension of ST was measured three-dimensionally as defined in Table 2. Landmarks were set on the reconstructed 3D models, and their positions were verified two-dimensionally on the axial, coronal and sagittal layers of the CTs/CBCTs (Table 3).

**Table 2:** Definition of linear and volumetric measurements

Measurement	Abbreviation	Definition
*Linear*		
Sella turcica length	ST depth	Median-sagittal length of mid sella extension
Sella turcica width	ST width	Distance between left and right interclinoid midpoints
Sella turcica height	ST height	Median-sagittal height of caudo-cranial sella extension
Sella turcica diameter	ST diameter	Distance between most posterior cranial point in region of dorsum sellae and tuberculum sellae in median-sagittal plane
Cranial Base	CB	Distance between midpoint of sella and nasofrontal suture
*Volumetric*		
Total Volume	Vol_T	Total volume in mililiter of the reconstructed region
Volume Anterior	Vol_A	Anterior volume of the complete volume in mililiter of the reconstructed region located anterior to the sectional plane of the posterior clinoid process and sella base
Volume Posterior	Vol_P	Posterior volume of the total volume in mililiter of the reconstructed region located at the sectional plane of the posterior clinoid process and sella base

**Table 3:**
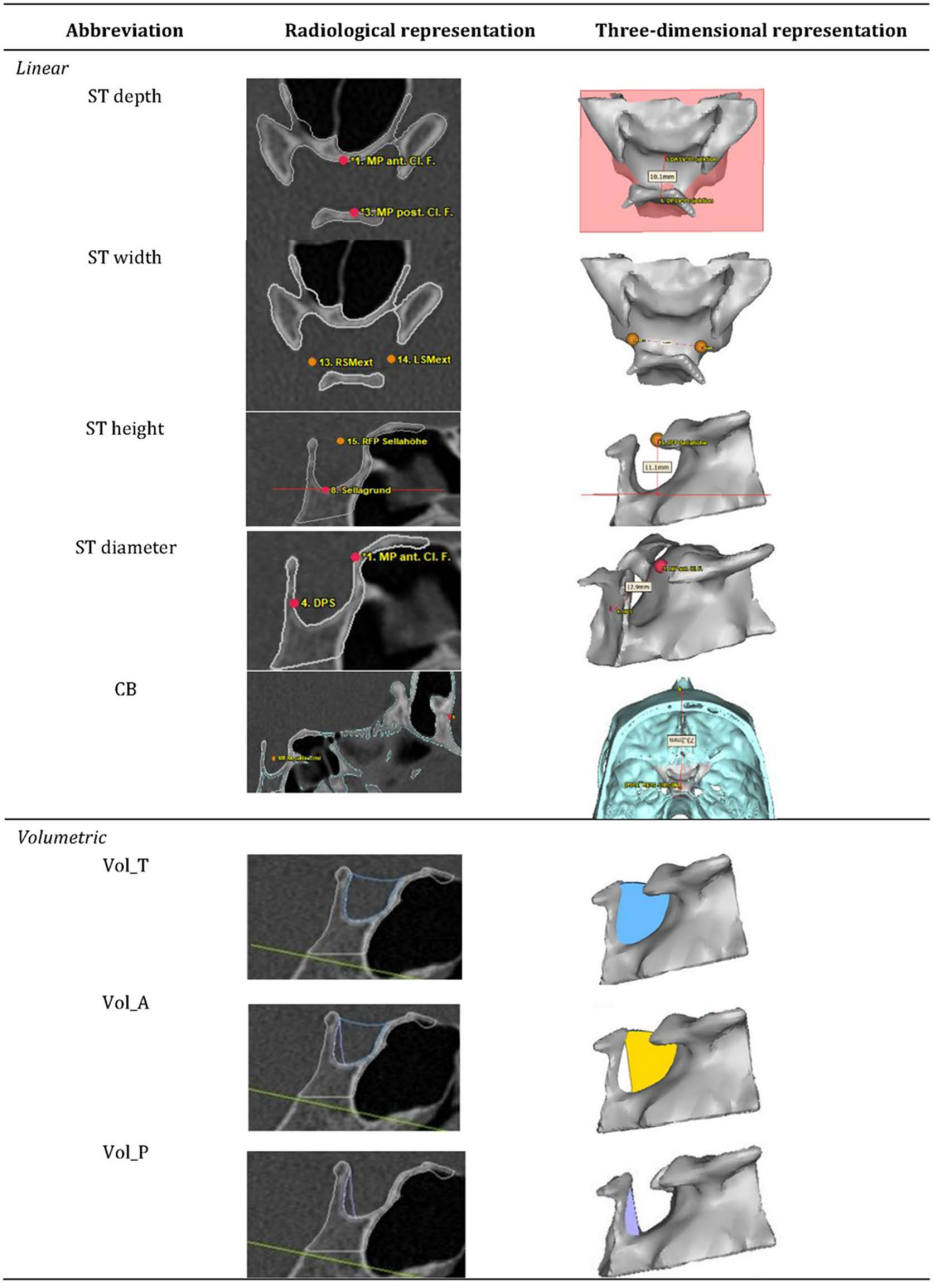
Illustration of linear and volumetric measurements

**Fig. 1:**
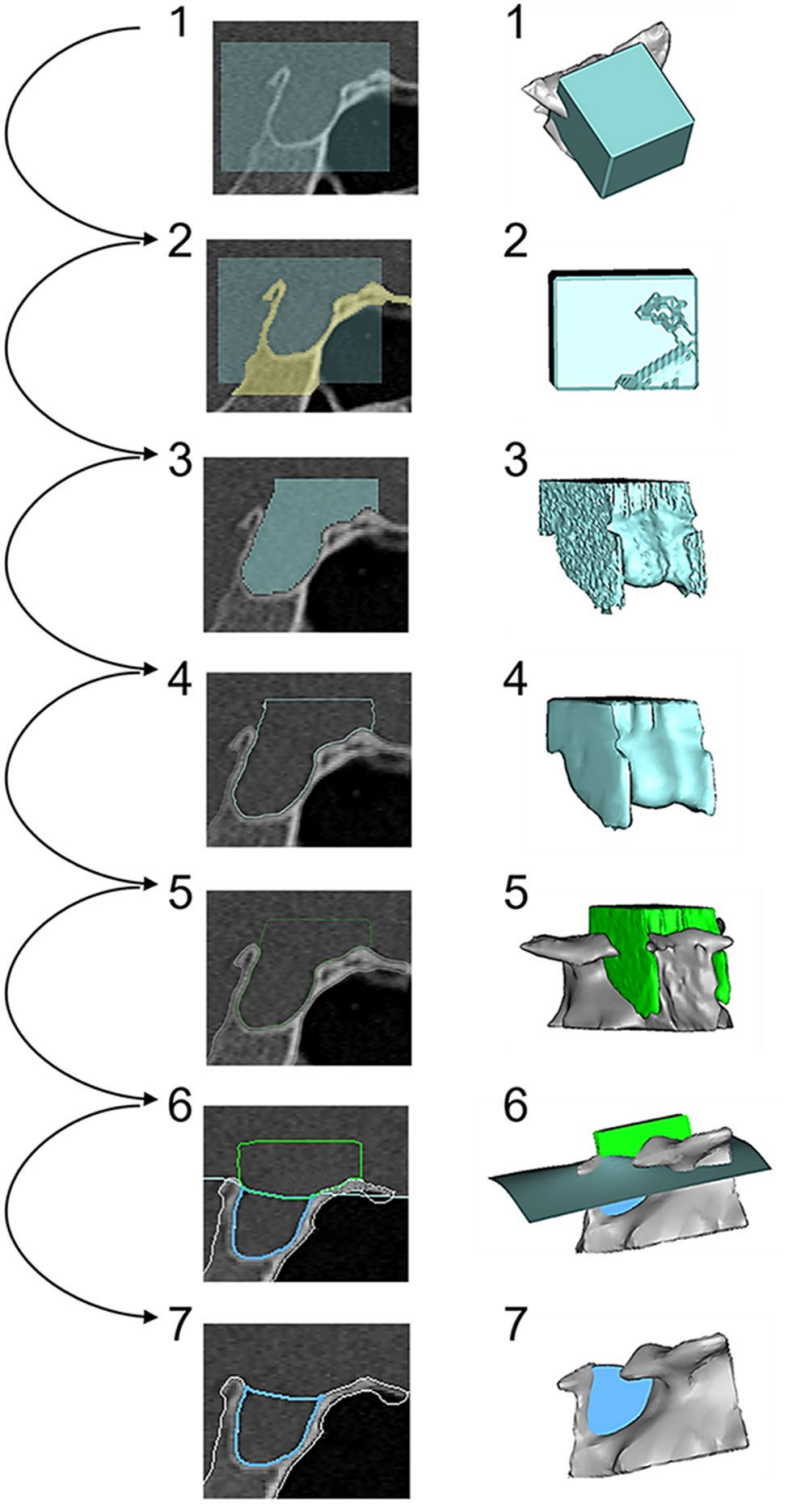
Stepwise 3D reconstruction of the sella turcica (ST) from CT/CBCT data. The eight steps show (**1**) standardized segmentation cube, (**2**) subtraction of cranial base, (**3**) raw ST model, (**4–6**) threshold adjustments and segmentation refinement, (**7**) application of cranial cutting plane, and (**8**) finalized volumetric model. Right side: corresponding 3D surface model. Left side: axial-sagittal image planes. All reconstructions were performed using Mimics software (Materialise, Leuven, Belgium)

To calculate ST volume, a segmentation process was developed using Mimics inPrint 3.0 and MIS 25.0 (Materialise, Leuven, Belgium) within the threshold of 2047 to -728 units (Fig. 1). The applied threshold refers to grayscale units (GU) as defined by the Mimics software. These values are not calibrated in Hounsfield units (HU) but represent internal intensity values optimized for bone segmentation in clinical CT and CBCT datasets. This software-specific thresholding approach ensures consistent segmentation results within the given spatial resolution and contrast limitations of the imaging modalities used [19]. The fixed range was applied uniformly across all patient datasets to ensure comparability. The upper and lower threshold boundaries were confirmed through preliminary visual inspection in multiple planes (axial, coronal, sagittal) to verify appropriate bone-to-soft tissue separation [19]. Threshold integrity was further validated using the porcine skull models, where segmentation accuracy could be compared to high-resolution micro-CT reference volumes.

**Fig. 2:**
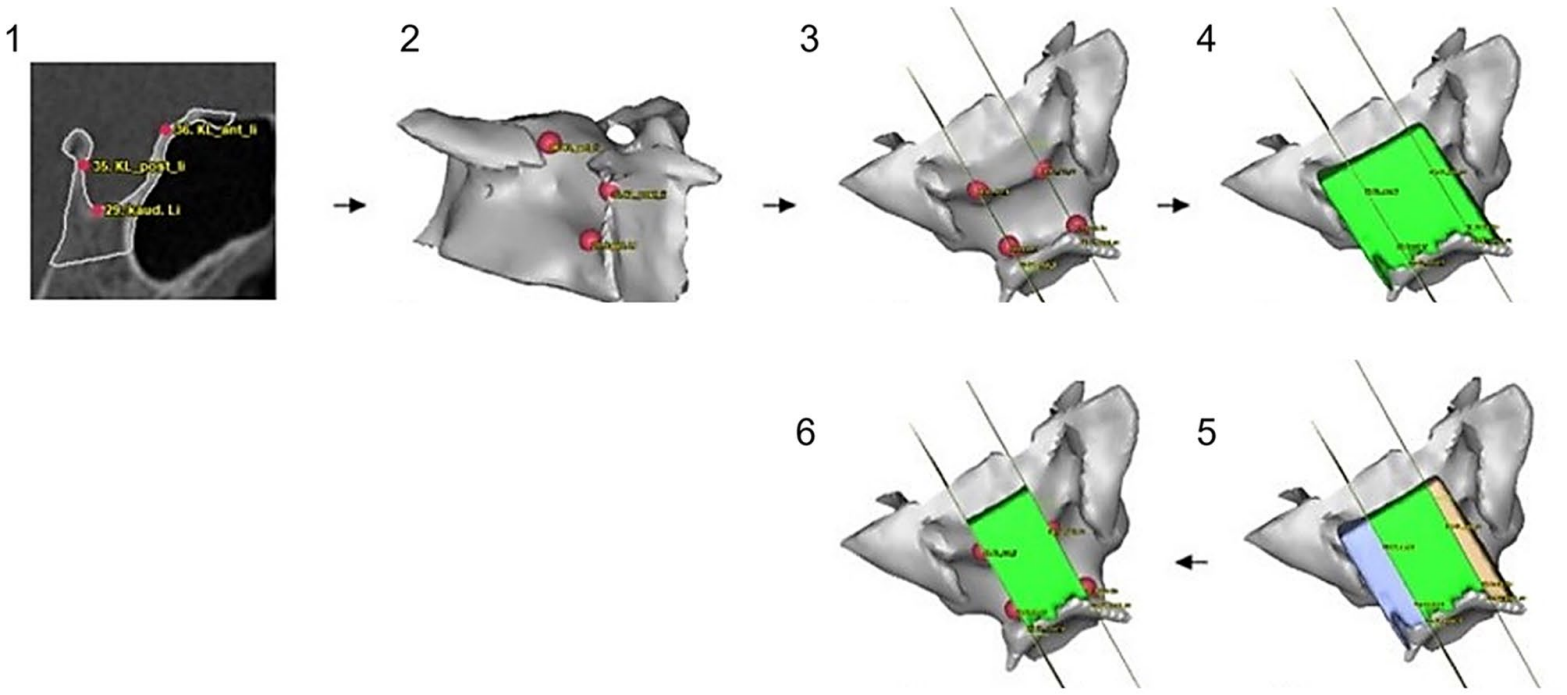
Representation of lateral ST limitation within 3D reconstruction. (**1**) radiological identification of segmentation points, (**2**) control of bilateral representation in reconstruction, (**3**) creation of lateral segmentation planes between the most caudo-lateral region; the most cranio-anterior-lateral region and the most cranio-posterior-lateral region, (**4**) insertion of vertically finalized ST model, (**5**) segmentation of lateral boundaries, (**6**) laterally finalized ST model

Irrelevant parts of the cranial base were removed using software-integrated subtraction tools with voxel-level precision (0.3 mm). For volume reconstruction of the ST, a standardized cube exceeding the region of interest was placed over the relevant area in all cases. From this cube, the osseous boundaries of the ST were segmented to create a positive model. The stepwise segmentation process and definition of lateral and cranial boundaries are illustrated in Figs. 1 and 2, respectively. These figures provide a visual overview of how the osseous and non-ossified borders were defined and reconstructed during the modeling process. Since the lateral and cranial margins of the ST are not fully ossified, these non-bony borders were closed using planar surfaces, each defined by three reproducible anatomical landmarks (Figs. 1 and 2). The resulting volume model was then trimmed along the reconstructed planes using a cutting thickness of 0.1 mm.

**Fig. 3:**
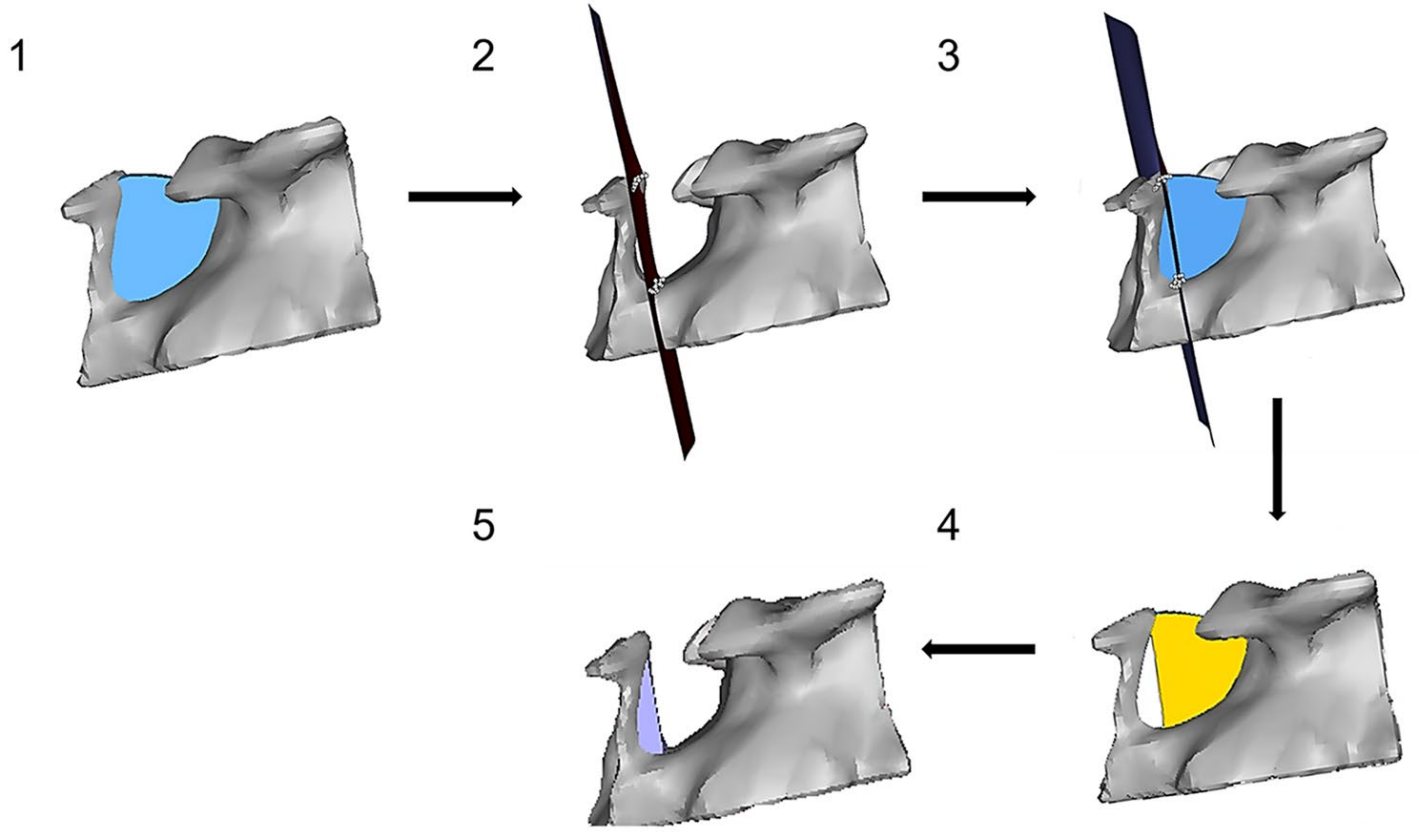
Representation of lateral ST limitation within 3D reconstruction. (**1**) radiological identification of segmentation points, (**2**) control of bilateral representation in reconstruction, (**3**) creation of lateral segmentation planes between the most caudo-lateral region; the most cranio-anterior-lateral region and the most cranio-posterior-lateral region, (**4**) insertion of vertically finalized ST model, (**5**) segmentation of lateral boundaries, (**6**) laterally finalized ST model

After subtraction of cranial and lateral parts, the total volume (Vol_T) was calculated. The segmentation in anterior and posterior ST volume (Vol_A and Vol_P) was performed along the plane defined by the posterior clinoid process, dorsum sellae and caudal extension of the ST floor (Fig. 3). Vol_A was calculated by subtracting Vol_P from Vol_T (Fig. 3).

### Validation

To assess the imaging accuracy of CT and CBCT scans, five decapitated porcine skulls were examined using both modalities under the imaging parameters described above. As ex-vivo gold standard, high-resolution micro-computed tomography (micro-CT) was performed (Quantum FX, PerkinElmer Inc., USA) with the following parameters: tube voltage 90 kV, tube current 200 microamperes (µA), field of view 73 × 73 mm, exposure time 2 min. Data were reconstructed on a 512 × 512 matrix, resulting in a pixel size of 143 μm. Phantom grid scans confirmed an isotropic physical resolution of approximately one pixel, and a resolution of 150 μm was therefore assumed. This resolution was substantially higher than that of conventional CT and CBCT systems. Prior to micro-CT scanning, the mandible and maxilla were separated from the skull to isolate the region of interest. The remaining cranial segment was positioned in the scanner with the calvaria oriented downward to ensure complete coverage of the target area.

**Fig. 4:**
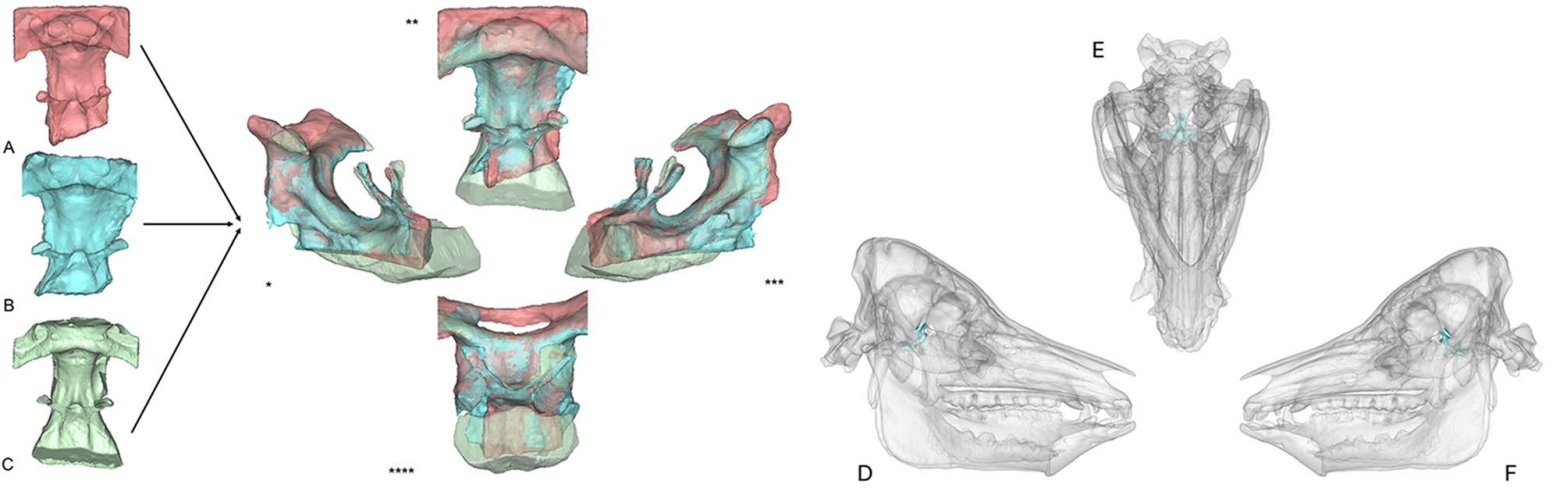
Superimposition. Illustration of ST reconstruction from the same porcine specimen based on different imaging techniques: CBCT (**A**), CT (**B**), micro-CT (**C**). Views: dorso-sagittal left (*),cranial (**),dorso-sagittal right (***),dorsal (****). Reconstruction of ST models in porcine skull from right-lateral (**D**), axial (**E**) and left-lateral (**F**)

Following the same methodology as described above, volumetric measurements of the porcine STs were performed using micro-CT, CBCT, and CT imaging. For micro-CT data, minimum and maximum threshold values were set at 600 and 2697 grayscale units, respectively. As micro-CT lacks standardized Hounsfield unit calibration, thresholds were defined individually for each skull based on the mean value between the average intensity of bone and that of the surrounding soft tissue. All volume measurements were calculated in cubic millimeters (mm³). In total, three 3D models of the ST and its volume for each porcine skull were analyzed (Fig. 4).

### Statistical analysis

Descriptive statistics and analyses were performed using SPSS (v. 30, IBM, New York, USA). Data was assumed as non-normally distributed. Intra- and interrater agreements of all measurements were assessed by Bland-Altman-plots (B&A plots). The porcine sella volume was compared between CBCT and microCT as well as between CT and microCT using B&A plots. The linear and volumetric measurements characterizing sella turcica morphology were analyzed according to skeletal classes, vertical relations, symmetry and gender by Kruskal-Wallis-test. For skeletal classes and vertical relations Kruskal-Wallis-test was followed up with Bonferroni post-hoc tests if appropriate. Correlation between the anterior volume and the Wits Appraisal was tested using Spearman’s-Rho. α-level was set at 0.05. A sample size calculation using G*Power 3.1 [24] based on α = 0.05 (two-sided) and power of 1 − β = 0.90 was performed. Values for ST lengths were taken from the literature [25]: Class I Mean 7.92, SD 0.84; Class II Mean 8.48, SD 0.30. An effect size (Cohen’s d) of 0.89 was calculated. These values suggested that each group required a minimum of 28 participants. Considering a potential dropout rate, the sample size was adjusted to 30 participants per group.

## Results

In total, the ST of 90 participants was evaluated. B&A plots revealed high intra- and interrater agreements for linear measurements (Table 4). All measurements were within the limits of agreement.

**Table 4:** Assessment of intra- and interrater agreement by B&A plots revealed reliable and clinical acceptable measurements

Measurement	Agreement	M_Diff_	SD	LoA+	LoA-
ST length					
	Intra	0.02	0.14	0.29	-0.25
	Inter	-0.04	0.17	0.29	-0.37
ST width					
	Intra	0.02	0.17	0.35	-0.31
	Inter	-0.05	0.2	0.34	-0.44
ST height					
	Intra	-0.01	0.09	0.17	-0.19
	Inter	0.03	0.11	0.25	-0.19
ST diameter					
	Intra	0.01	0.21	0.42	-0.40
	Inter	0.06	0.15	0.35	-0.23
CB					
	Intra	-0.07	0.39	0.69	-0.83
	Inter	0.03	0.19	0.40	-0.34

ST = sella turcica, M_Diff_=mean difference, SD = standard deviation, LoA + = upper limit of agreement, LoA-=lower limit of agreement

The mean difference of the porcine ST volume between CBCT and microCT was − 37.32 mm^3^ (SD = 39.77 mm3), which means the mean ST volume measured by CBCT was 8.7% smaller than the volume measured by microCT. All measurements were within the limits of agreement according to B&A plots. For CT, the mean volumetric difference was − 39.19 mm^3^ (SD = 36.28 mm3). B&A plots demonstrated that all measurements were within the limits of agreement even though the CT volumes were on average 10% smaller. These results emphasize that although CT and CBCT provide sufficiently reliable measurements for orthodontic purposes, their tendency to underestimate true ST volume should be considered when evaluating patients for pituitary-related pathologies, where small volumetric changes may have diagnostic significance [6, 19].

Standardized segmentation of the lateral and cranial ST borders, as illustrated in Fig. 2, ensured consistency in volumetric analysis across all skeletal classes. While skeletal class had no impact on ST width, -height, -diameter and cranial base (Table 5), ST length differed significantly between the skeletal classes. Participants with skeletal class I showed higher ST length than individuals with skeletal class II (post-hoc test: *p* = .024). ST length of participants with skeletal class III was similar to ST length of participants with class II (post-hoc test: *p* = .387) or class I (post-hoc test: *p* = .764).

**Table 5:** Median (*Mdn*) and interquartile range (IQR) of Sella turcica (ST) morphology characteristics according to skeletal classes

	Class I (*n* = 30)		Class II (*n* = 30)		Class III (*n* = 30)		*p*-value
	Mdn	IQR	Mdn	IQR	Mdn	IQR	
ST length (mm)	10.6	3.0	9.6	1.9	10.1	2.6	0.029*
ST width (mm)	11.2	2.3	11.0	1.5	11.0	1.7	0.808
ST height (mm)	7.9	1.3	8.4	1.4	7.7	1.4	0.592
ST diameter (mm)	12.5	2.3	12.0	2.0	11.9	1.8	0.252
CB (mm)	71.2	6.5	68.6	4.9	67.9	5.3	0.065
Vol_T (mm^2^)	932.2	269.3	996.7	193.9	852.3	269.1	0.049*
Vol_A (mm^2^)	719.6	162.9	784.9	199.5	646.4	239.1	0.024*
Vol_P (mm^2^)	183.2	68.3	220.8	150.9	194.6	98.9	0.442

* Significant result according to Kruskal-Wallis-test. ST = sella turcica, Vol_T = total sella volume, Vol_A = anterior sella volume, Vol_P = posterior sella volume

The total ST volume was significantly higher in subjects with skeletal class II than in participants with class III (post-hoc test: *p* = .047). No difference was observed between class II and class I (post-hoc test: *p* = .348) or class III and class I (post-hoc test: *p* = 1.0). While the posterior volume was similar between the classes, the anterior volume was higher in class II than class III (post-hoc test: *p* = .019). Again, no difference between class II and class I (post-hoc test: *p* = .427) or class III and class I (post-hoc test: *p* = .623) was found. The anterior volume was significantly related to the Wits appraisal (*r* = .337; *p* < .001), suggesting a structural relationship between the anterior ST architecture and the sagittal jaw relationship.

Neither vertical relation (Table 6) nor symmetry (Table 7) affected ST morphology significantly.

**Table 6:** Median (*Mdn*) and interquartile range (IQR) of Sella turcica (ST) morphology characteristics according to vertical jaw relationship

	Hypodivergent (*n* = 30)		Neutral (*n* = 28)		Hyperdivergent (*n* = 32)		*p*-value
	Mdn	IQR	Mdn	IQR	Mdn	IQR	
ST length (mm)	10.4	2.3	9.8	1.9	9.9	3.0	0.59
ST width (mm)	11.4	2.5	11.0	1.6	11.1	1.7	0.871
ST height (mm)	7.8	1.5	7.9	1.6	8.1	1.6	0.344
ST diameter (mm)	12.1	2.3	12.1	1.5	12.1	2.2	0.997
CB (mm)	69.0	7.2	70.2	5.0	68.0	5.6	0.116
Vol_T (mm^2^)	880.5	314.0	923.1	232.4	974.8	266.8	0.231
Vol_A (mm^2^)	652.4	232.0	719.6	141.7	797.8	245.1	0.142
Vol_P (mm^2^)	186.8	129.5	176.1	131.9	214.1	92.3	0.391

ST = sella turcica, Vol_T = total sella volume, Vol_A = anterior sella volume, Vol_P = posterior sella volume

**Table 7:** Median (*Mdn*) and interquartile range (IQR) of Sella turcica (ST) morphology characteristics according to symmetry

	Symmetric (*n* = 57)		Asymmetric (*n* = 33)		*p*-value
	Mdn	IQR	Mdn	IQR	
ST length (mm)	10.1	2.1	9.8	2.3	0.236
ST width (mm)	11.4	1.7	10.8	1.6	0.167
ST height (mm)	7.9	1.4	7.7	1.4	0.283
ST diameter (mm)	12.2	2.3	11.7	1.8	0.18
CB (mm)	69.1	6.8	68.0	5.0	0.395
Vol_T (mm^2^)	940.6	242.1	927.5	264.0	0.99
Vol_A (mm^2^)	720.8	219.3	715.2	213.7	0.709
Vol_P (mm^2^)	184.6	125.9	198.2	120.3	0.549

ST = sella turcica, Vol_T = total sella volume, Vol_A = anterior sella volume, Vol_P = posterior sella volume

Gender-related differences were only found in the position of the ST relative to the nasofrontal suture (CB) (*p* < .001), while other ST parameters remained independent of gender. These findings support the relevance of ST morphology as a potential indicator of skeletal malocclusion, particularly in the sagittal context, and confirm the focus of the analysis on structural parameters in the sella turcica region. (Table 8). ST length, width, height and diameter as well as sella volumes did not differ between men and women.

**Table 8:** Median (*Mdn*) and interquartile range (IQR) of Sella turcica (ST) morphology characteristics according to gender

	Men (*n* = 44)		Women (*n* = 46)		*p*-value
	Mdn	IQR	Mdn	IQR	
ST length (mm)	10.0	2.1	9.9	2.6	0.406
ST width (mm)	10.9	2.0	11.2	1.7	0.379
ST height (mm)	7.8	1.7	8.0	1.3	0.385
ST diameter (mm)	12.1	2.7	12.1	1.8	0.812
CB (mm)	71.8	4.6	67.0	3.7	< 0.001*
Vol_T (mm^2^)	930.3	255.9	922.3	251.8	0.784
Vol_A (mm^2^)	723.3	207.1	717.3	221.8	0.923
Vol_P (mm^2^)	194.4	85.7	190.4	138.1	0.41

* Significant result according to Kruskal-Wallis-test ST = sella turcica, Vol_T = total sella volume, Vol_A = anterior sella volume, Vol_P = posterior sella volume

## Discussion

The present study provides novel insights into the morphological characteristics of the ST and their variation in relation to maxillomandibular configuration and gender. The findings are in line with previous studies that have analyzed ST morphology using different imaging modalities such as LC, CBCT and CT scans [6, 7, 11, 15, 25, 26, 27]. The results support the general applicability of CBCT and CT for volumetric analysis of the ST, while also highlighting measurable discrepancies when compared to high-resolution micro-CT data.

The investigation revealed a high intra- and inter-rater agreement for ST measurements. However, both CBCT and CT showed a tendency to underestimate ST volume relative to micro-CT. These deviations can likely be attributed to the lower spatial resolution and contrast differentiation of clinical imaging systems, as well as limitations in segmentation accuracy. This may be of particular relevance in endocrinological contexts, where precise volumetric assessment of the ST is critical, especially for the diagnosis and monitoring of pituitary pathologies [26].

The core result of this study is the difference in ST length between the skeletal classes. Participants with skeletal Class I exhibited longer STs compared to those with Class II (*p* = .024), whereas no significant differences were observed between Class III and either Class I or II. This finding partially aligns with the results reported by Alkofide [6], who also noted associations between skeletal classification and ST morphology. While ST width, height, and diameter were unaffected by skeletal class, ST length discriminated class I individuals from class II individuals.

This contrasts with the meta-analysis by Gong et al. [28], which reported significantly shorter anterior and total cranial base lengths as well as smaller cranial base angles (NSBa and NSAr) in class III compared to class I and II. The authors interpreted these findings as structural shortening and flattening of the anterior skull base in Class III malocclusions - a correlation that could indicate an accompanying reduction in ST length.

However, the lack of ST length shortening observed in our study can be explained by several factors. First, the meta-analysis by Gong et al. [28] based on 2D data and classic linear reference distances (e.g., N–S–Ba), whereas our study recorded the ST as an isolated 3D structural element. The ST length in our design is therefore a purely local measure and is not necessarily directly comparable with the anterior base length in the classic sense. It is therefore conceivable that skeletal differences in Class III tend to manifest themselves in angular characteristics and extensive basal structures, while ST length remains relatively stable as a local measure.

Second, there was increased variance in ST lengths within the Class III group, indicating greater morphological heterogeneity in this patient group. This wide variation could level out differences that would be significant in more homogeneous subgroups. In addition, the absence of ST shortening could be masked by compensatory growth mechanisms, such as dorsal expansion of the sella region in the context of mandibular prognathism.

Third, although no significant length differences were found in Class III, there was a significantly reduced anterior ST volume component compared to Class II. This suggests that volumetric or segmental features may be more sensitive to skeletal patterns than one-dimensional measurements. In this respect, the integration of volumetric, angle-based, and shape-oriented analyses could enable a more differentiated assessment of Class III-associated changes in the future. This literature comparison suggests that ST length measurements, when considered in isolation, do not necessarily correspond to known cranial base shortening in Class III. Rather, a differentiated analysis incorporating volumetric and angle-dependent parameters appears to be necessary in order to adequately reflect the complex interaction between ST morphology and sagittal malocclusion.

Furthermore, total ST volume was significantly greater in class II participants compared to class III, which was mainly due to differences in anterior ST volume. These findings are consistent with previous studies that emphasize ST variation in individuals with distinct craniofacial patterns [7, 11]. The moderate correlation observed between anterior ST volume and the Wits appraisal further supports the influence of sagittal skeletal configuration on ST morphology. One possible biological explanation lies in the anatomical and functional proximity of the anterior sella turcica to the pituitary gland, which plays a key role in regulating somatic and craniofacial growth. Variations in anterior ST volume may reflect differences in local bone development or pituitary-related growth activity, particularly during key developmental phases. This could be especially relevant in skeletal class II cases, where altered mandibular growth is a common feature [6, 15].

Clinically, this association may offer additional diagnostic value in borderline cases or early treatment planning. If future longitudinal studies confirm the stability and predictive value of anterior ST volume, it could be integrated into 3D diagnostic protocols as a supplementary marker for skeletal class assessment or growth prognosis. Such a marker would be particularly useful in pediatric or adolescent patients, where treatment timing is critical.

In contrast, no significant differences in ST morphology were found across vertical skeletal patterns or between symmetrical and asymmetrical facial configurations. This could be explained by the central position and relative stability of the sella turcica within the cranial base, which may be less susceptible to alterations driven by vertical or transverse growth trajectories [5, 29]. Additionally, the linear and volumetric measurements applied in this study may not be sensitive enough to capture more subtle shape deviations associated with vertical dysplasia or mild asymmetries [16, 30]. From a clinical perspective, this suggests that ST morphology may hold more relevance in the context of sagittal skeletal evaluation, while its utility in assessing vertical or transversal discrepancies appears limited. Future studies employing more refined morphometric or shape-based analysis methods may provide further insight.

Male participants exhibited significantly greater distances between the sella turcica (ST) and the fronto-nasal suture compared to female participants. In contrast, no significant sex-related differences were observed in ST length, width, height, diameter, or volume. These findings are in line with the meta-analysis by Iskra et al. [25], which reported that while gender-related differences in ST morphology exist, they do not consistently affect all anatomical dimensions. Recognizing such variations is essential for accurately distinguishing between normal anatomical diversity and potential pathological alterations involving the ST.

ST morphology is increasingly recognized as a diagnostic marker for pituitary diseases, craniofacial- [18] and dental anomalies [13, 20, 27]. The presented results emphasize the importance of careful interpretation of ST measurements in relation to the imaging modality, skeletal classification, and patient demographics. Although CBCT and CT provide reliable measurements, slight underestimates should be considered in volumetric assessments. Furthermore, the observed associations between ST morphology and skeletal classification underscore the potential role of ST analysis in orthodontic research. Future research should investigate longitudinal changes in ST morphology in different age groups and possible correlations with endocrine disorders. The inclusion of advanced imaging techniques such as MRI could provide further insights into the soft tissue structures surrounding the ST and complement the existing radiological investigations.

Beyond orthodontics, our findings have implications for craniofacial and endocrine imaging. The systematic underestimation of ST volume by CT and CBCT, as compared to micro-CT, highlights limitations in the diagnostic accuracy of clinical imaging - particularly relevant in evaluating pituitary-related pathologies such as adenomas or hypoplasia [6, 19]. These deviations should be considered when interpreting ST size in endocrine or radiological contexts [25]. In orthodontics, the correlation between anterior ST volume and sagittal skeletal class supports the use of 3D ST analysis as a potential supplementary marker in assessing jaw discrepancies [15, 16]. Future studies should explore its prognostic value in early treatment planning, particularly in growing patients.

### Limitations

Several limitations of the present study should be considered. First, although CT and CBCT provide valuable three-dimensional information, their spatial resolution remains inferior to that of micro-CT. As a result, small anatomical structures and fine bony contours may be underestimated due to partial volume effects and lower contrast sensitivity. This could have influenced the accuracy of the volumetric measurements.

Second, the segmentation of non-ossified boundaries required semi-automated reconstruction using planar surfaces defined by reproducible anatomical landmarks. While this approach is necessary to reconstruct the ST as a closed volume, it introduces a potential source of subjectivity. Even though all reconstructions were performed following a standardized protocol and verified by two independent investigators, inter-operator variability cannot be entirely ruled out. Previous studies have shown that the ST can be identified with relatively high consistency in CBCT datasets [29], and that the use of landmark-based reference systems improves reproducibility [5]. However, the inherent anatomical variability of the sella turcica [25] emphasizes the need for cautious interpretation of absolute volumetric data.

Third, the study design was cross-sectional and did not include longitudinal data. Therefore, conclusions regarding developmental changes or age-related remodeling of the ST remain limited.

Additionally, the sample was restricted to adult patients with distinct dentofacial anomalies, which may limit the generalizability of the findings to broader populations, including individuals without malocclusion or those with syndromic conditions.

Finally, although micro-CT was used as a high-resolution reference in the in vitro analysis, the porcine model may not fully reflect the morphological complexity of the human ST. Species-specific anatomical differences should therefore be considered when interpreting the comparative results.

## Conclusions

This study provides new insights into the 3D morphology of ST in relation to dentofacial anomalies. While CT and CBCT proved to be suitable tools for volumetric assessment, a systematic underestimation was found compared to micro-CT. Morphological differences in ST length and volume were associated with dentofacial anomalies, particularly between Class I and Class II participants, with anterior ST volume showing a moderate correlation with the Wits score. These findings suggest that ST morphology may reflect certain aspects of sagittal skeletal discrepancy in adults. However, due to the cross-sectional nature of the study, direct implications for the development of dentofacial anomalies must be interpreted with caution. Future longitudinal studies are needed to assess whether the anterior ST volume could serve as a stable anatomical marker or risk indicator for the early detection of dentofacial anomalies.

## Data Availability

No datasets were generated or analysed during the current study.
